# Supplementary material for: Structural basis for the inhibition of IAPP fibril formation by the co-chaperonin prefoldin
Source: Nat Commun. 2022 May 2;13:2363. doi: 10.1038/s41467-022-30042-y (PMC9061850; doi:10.1038/s41467-022-30042-y)
Supplement: Supplementary file 1 — Supplementary Information [file 41467_2022_30042_MOESM1_ESM.pdf]

Structural Basis for the Inhibition of IAPP Fibril Formation by the Co-Chaperonin Prefoldin

Authors: Ricarda Törner[1,#], Tatsiana Kupreichyk[2,3,#], Lothar Gremer[2,3,4], Elisa Colas Debled[1], Daphna Fenel[1], Sarah Schemmert[2], Pierre Gans[1], Dieter Willbold[2,3,4], Guy Schoehn[1], Wolfgang Hoyer[2,3,*], Jerome Boisbouvier[1,*]

Affiliation:

1 Univ. Grenoble Alpes, CNRS, CEA, Institut de Biologie Structurale (IBS), 71, Avenue des Martyrs, F-38044 Grenoble, France.
2 Institute of Biological Information Processing (IBI-7: Structural Biochemistry) and JuStruct: Jülich Center for Structural Biology, Forschungszentrum Jülich, 52425 Jülich, Germany
3 Institut für Physikalische Biologie, Heinrich-Heine-Universität Düsseldorf, 40225 Düsseldorf, Germany
4 Research Center for Molecular Mechanisms of Aging and Age-Related Diseases, Moscow Institute of Physics and Technology (State University), Dolgoprudny, Russia
# These authors contributed equally

* correspondence to be addressed to wolfgang.hoyer@hhu.de or jerome.boisbouvier@ibs.fr

Supplementary Information

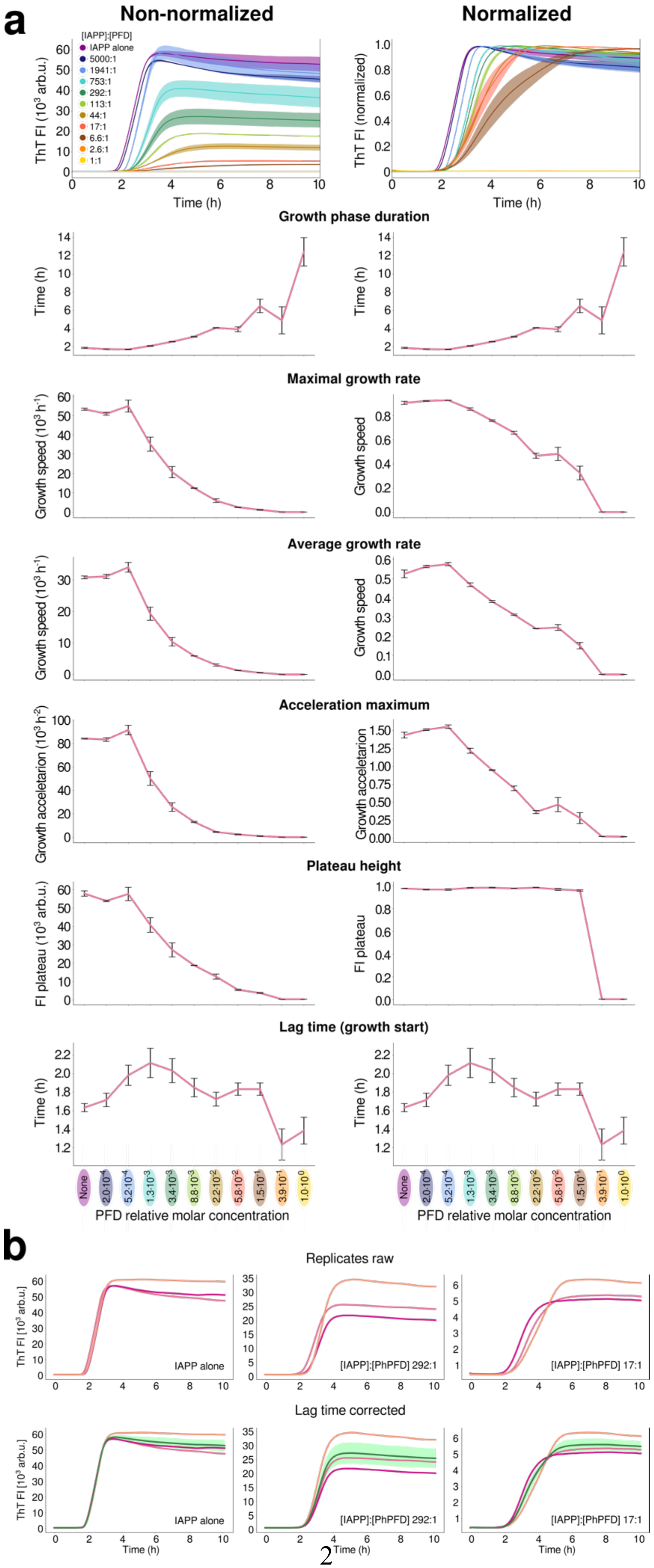
a
Non-normalized
Normalized
[IAPP]:[PFD]
IAPP alone
5000:1
1941:1
753:1
292:1
113:1
44:1
17:1
6.6:1
2.6:1
1:1
ThT FI (10³ arb.u.)
ThT FI (normalized)
Time (h)
Growth phase duration
Maximal growth rate
Growth speed (10³ h⁻¹)
Growth speed
Average growth rate
Acceleration maximum
Growth acceletarion (10³ h⁻²)
Growth acceletarion
Plateau height
FI plateau (10³ arb.u.)
FI plateau
Lag time (growth start)
None
2.0·10⁻⁴
5.2·10⁻⁴
1.3·10⁻³
3.4·10⁻³
8.8·10⁻³
2.2·10⁻²
5.8·10⁻²
1.5·10⁻¹
3.9·10⁻¹
1.0·10⁰
PFD relative molar concentration
b
Replicates raw
ThT FI [10³ arb.u.]
IAPP alone
[IAPP]:[PhPFD] 292:1
[IAPP]:[PhPFD] 17:1
Lag time corrected
Time (h)

Supplementary Figure 1: ThT assay kinetic analysis. a Comparison of kinetic parameters obtained for non-normalized (left) and normalized (right) data. The following parameters were extracted: growth phase duration, maximal growth rate, average growth rate, acceleration maximum, final plateau height, and lag time (growth start). The analysis illustrates that the concentration dependency trends are independent of data normalization, confirming the inhibitory effect of PFD on IAPP aggregation. Every curve in the top row represents a mean of n=3 replicates with the error shadows illustrating SD; the kinetic parameters are shown as mean ± SD. **b** Exemplary demonstration of raw triplicates and their appearance after the lag time correction as described in Methods section (averaged mean curves along with SD as error shades are shown in green). The presented analysis in (**a**) and (**b**) was done on the *de novo* IAPP (5 µM) aggregation kinetic assay in presence of PhPFD (also shown in Fig. 1a).

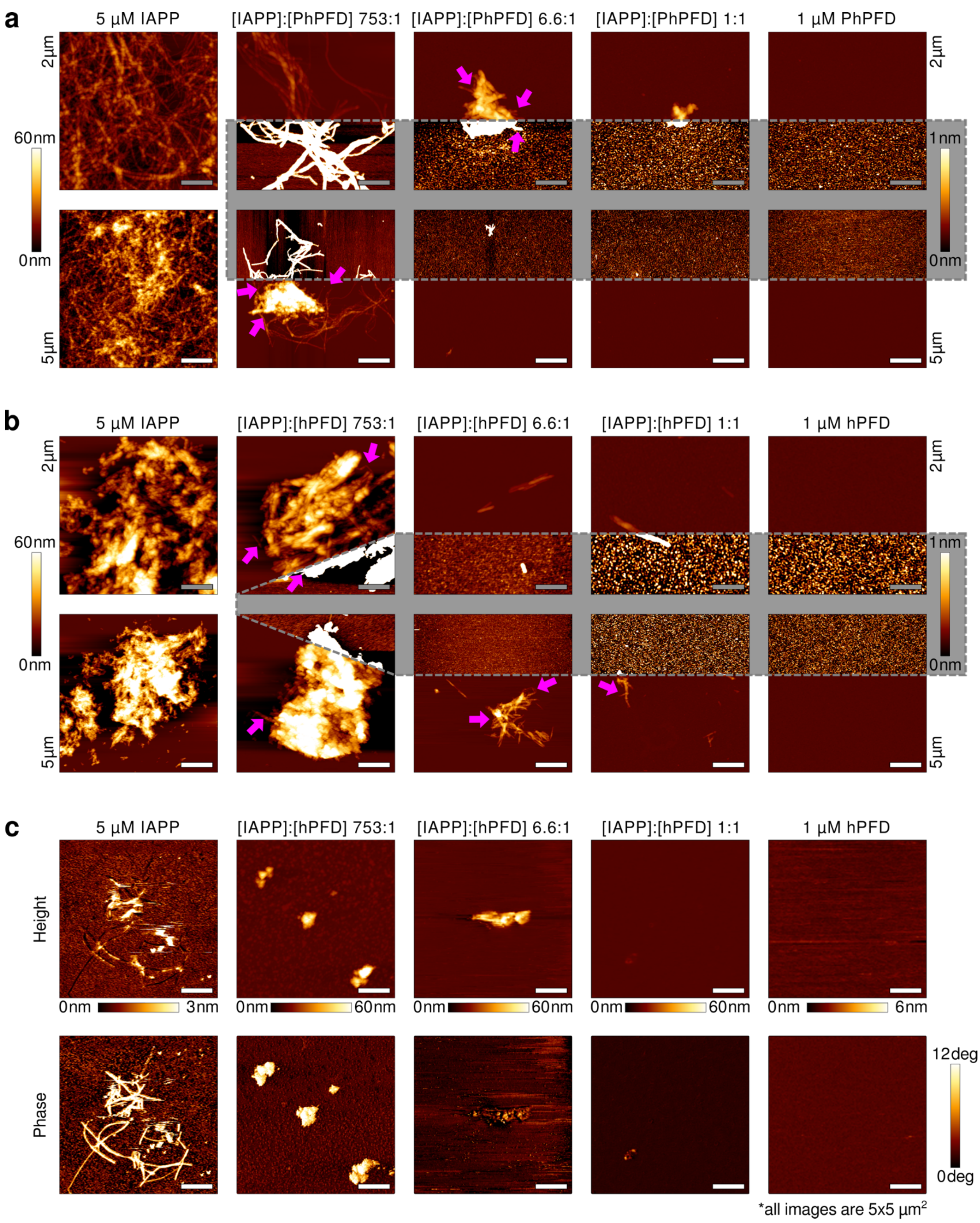
a
5 μM IAPP
[IAPP]:[PhPFD] 753:1
[IAPP]:[PhPFD] 6.6:1
[IAPP]:[PhPFD] 1:1
1 μM PhPFD
2μm
60nm
0nm
5μm
1nm
0nm
b
5 μM IAPP
[IAPP]:[hPFD] 753:1
[IAPP]:[hPFD] 6.6:1
[IAPP]:[hPFD] 1:1
1 μM hPFD
2μm
60nm
0nm
5μm
1nm
0nm
c
5 μM IAPP
[IAPP]:[hPFD] 753:1
[IAPP]:[hPFD] 6.6:1
[IAPP]:[hPFD] 1:1
1 μM hPFD
Height
Phase
0nm 3nm
0nm 60nm
0nm 60nm
0nm 60nm
0nm 6nm
12deg
0deg
*all images are 5x5 μm2

Supplementary Figure 2: Overview of IAPP aggregate morphology in presence of PFD. a and **b** AFM images of the samples after IAPP *de novo* aggregation assays in absence and presence of different concentrations of PhPFD (**a**) and hPFD (**b**) (respective kinetics are shown in Fig. 1a, top). Left to right: IAPP aggregated in absence of PFD; IAPP aggregates formed in presence of different concentrations of either PhPFD (**a**) or hPFD (**b**); prefoldin alone. The images show the close-ups of 2x2 μm^2 (top row) and overviews of 5x5 μm^2 (bottom row). For visualizing objects of diverse heights, two different colour code scaling were used: the gradient from dark brown to white represents either 0 nm to 60 nm height, or 0 nm to 1 nm (highlighted in grey). The arrows in magenta highlight individual fibrils sticking out of the bigger IAPP assemblies. Preparation of the samples for (**a**) and (**b**) included a drying step in order to assure the immobilization of the full set of different species present. **c** Height (top) and phase (bottom) 5x5 μm^2 AFM images performed in liquid confirming the tendencies reflected by the AFM images after a drying step (shown in (**a**) and (**b**)) and demonstrating that the clustered aggregates appear not due to sample drying, but they are present already in the solution.
Scale bars represent either 400 nm (grey) or 1 µm (white).

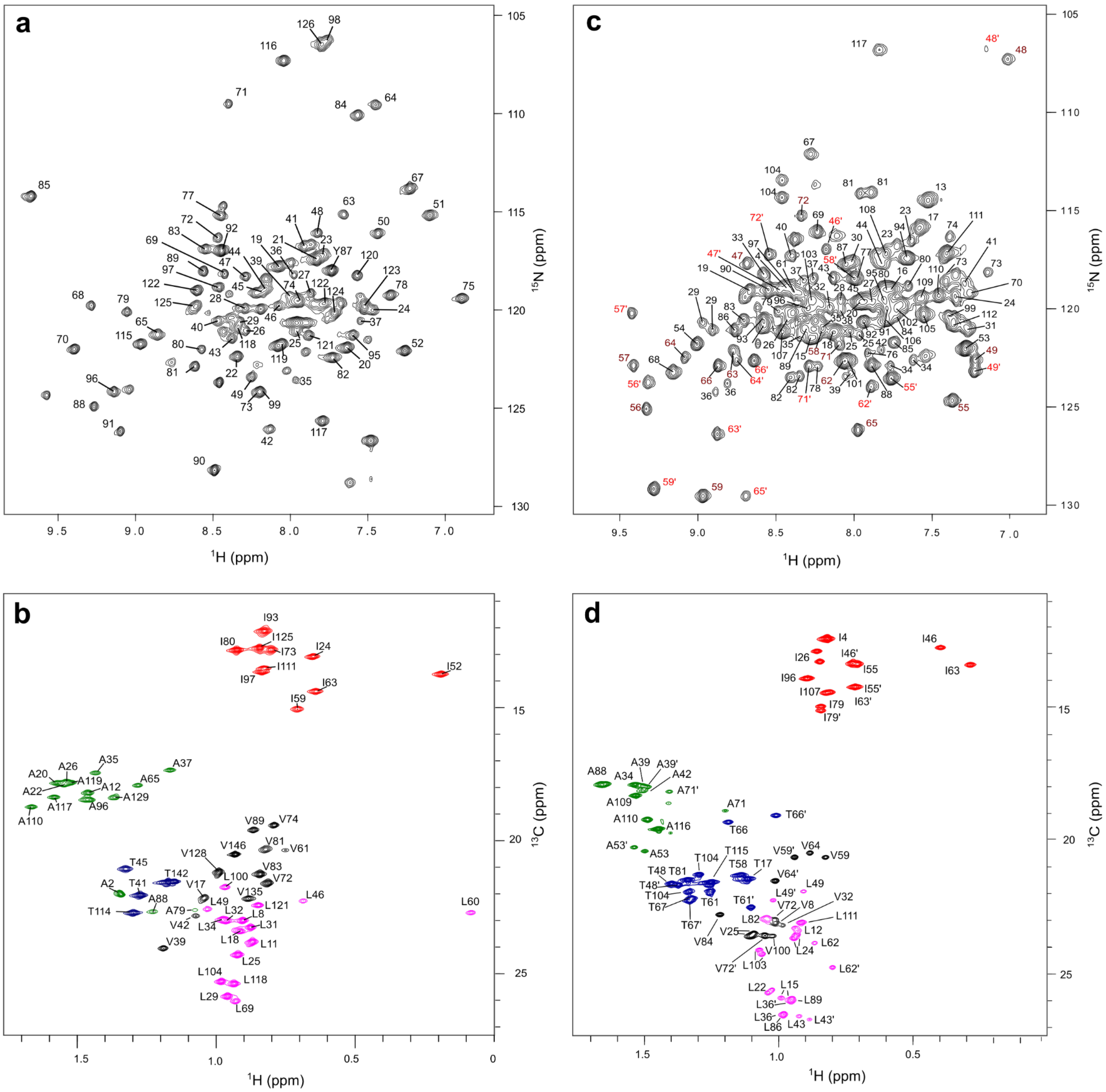

Supplementary Figure 3: Assigned 2D ^{15}N- and $^{13}CH_3$-TROSY spectra of PhPFD. NMR data were recorded at 70 °C on a NMR spectrometer operating at a ^{1}H frequency of 850 MHz. Assigned signals are annotated with corresponding residue numbers, and residues which could be assigned unambiguously to β/β' are annotated in red[45]. **a** 2D ^{15}N-TROSY spectrum of α−subunit. **b** 2D $^{13}CH_3$-TROSY spectrum of α-subunit. **c** 2D ^{15}N-TROSY spectrum of β−subunit. **d** 2D $^{13}CH_3$-TROSY spectrum of β-subunit. Colours designate different amino acid types (red: isoleucines, green: alanines, black: valines, blue: threonines, pink: leucines)

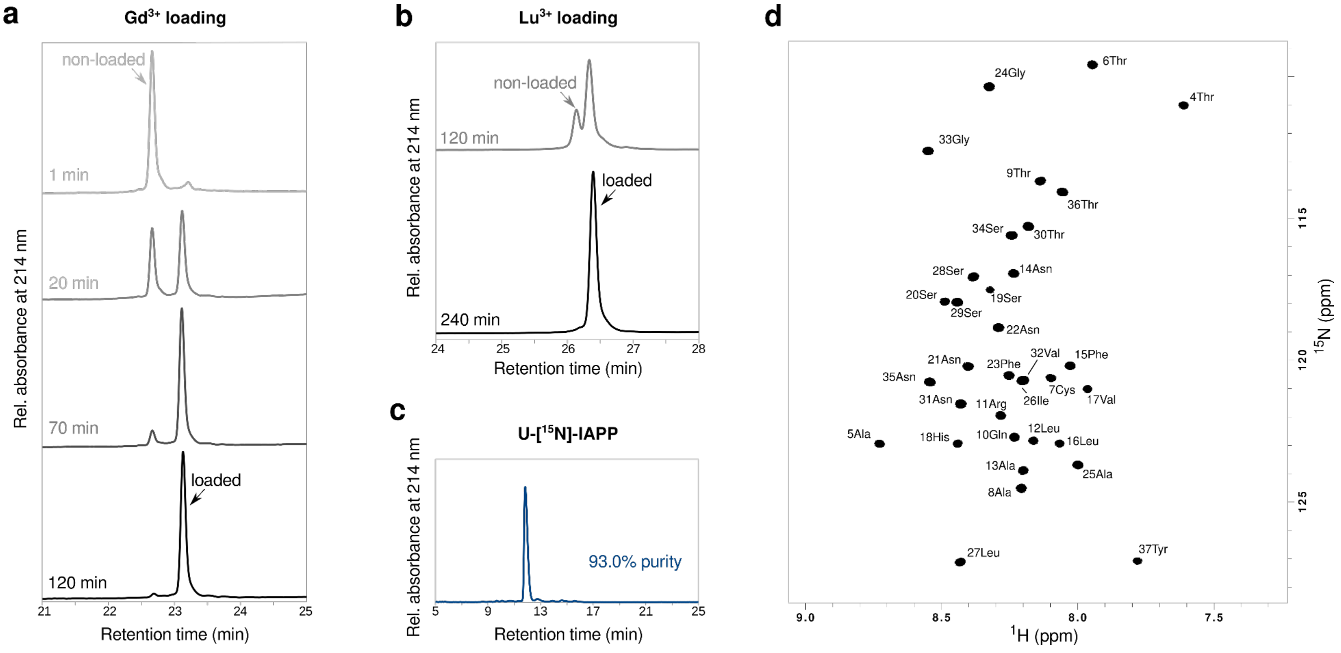

Supplementary Figure 4: IAPP quality control. Loading of DOTA-[β-Ala]-[β-Ala]-IAPP with Gd^{3+} (**a**) or Lu^{3+} (**b**) monitored by RP-HPLC at indicated incubation times. **c** Purity of recombinant U-[^{15}N]-IAPP by RP-HPLC. **d** 2D ^{15}N-TROSY spectrum of U-[^{15}N]-IAPP at 10 °C. List of assigned chemical shifts is available online (BMRB accession code 51259).

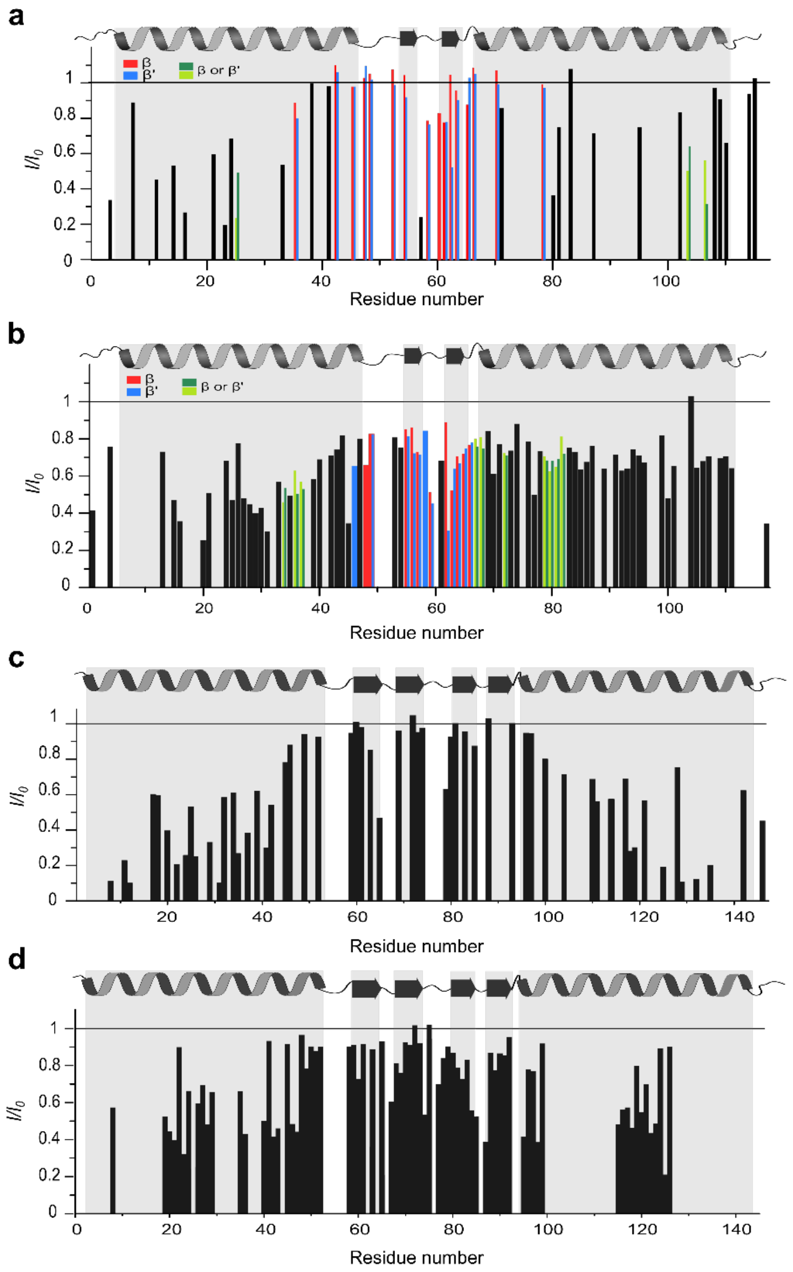

Supplementary Figure 5: Interaction between paramagnetically labelled IAPP and PhPFD. Histograms depicting I_{para}/I_{dia} intensity ratios of PhPFD signal detected using labelled PhPFD samples in presence of Gd-IAPP or Lu-IAPP, respectively. Reconstituted PhPFD with β subunits (**a**, **b**) and α subunits (**c**, **d**) labelled on $^{13}CH_3$ groups (**a**, **c**) or ^{15}NH-backbone groups (**b**, **d**) were mixed with Lu/Gd-loaded IAPP in a ratio of 1:2 ($^{13}CH_3$-labeling) or 1:1 (U-[^{15}N]-labelling). No values indicate missing assignment[45] or absence of methyl-groups.

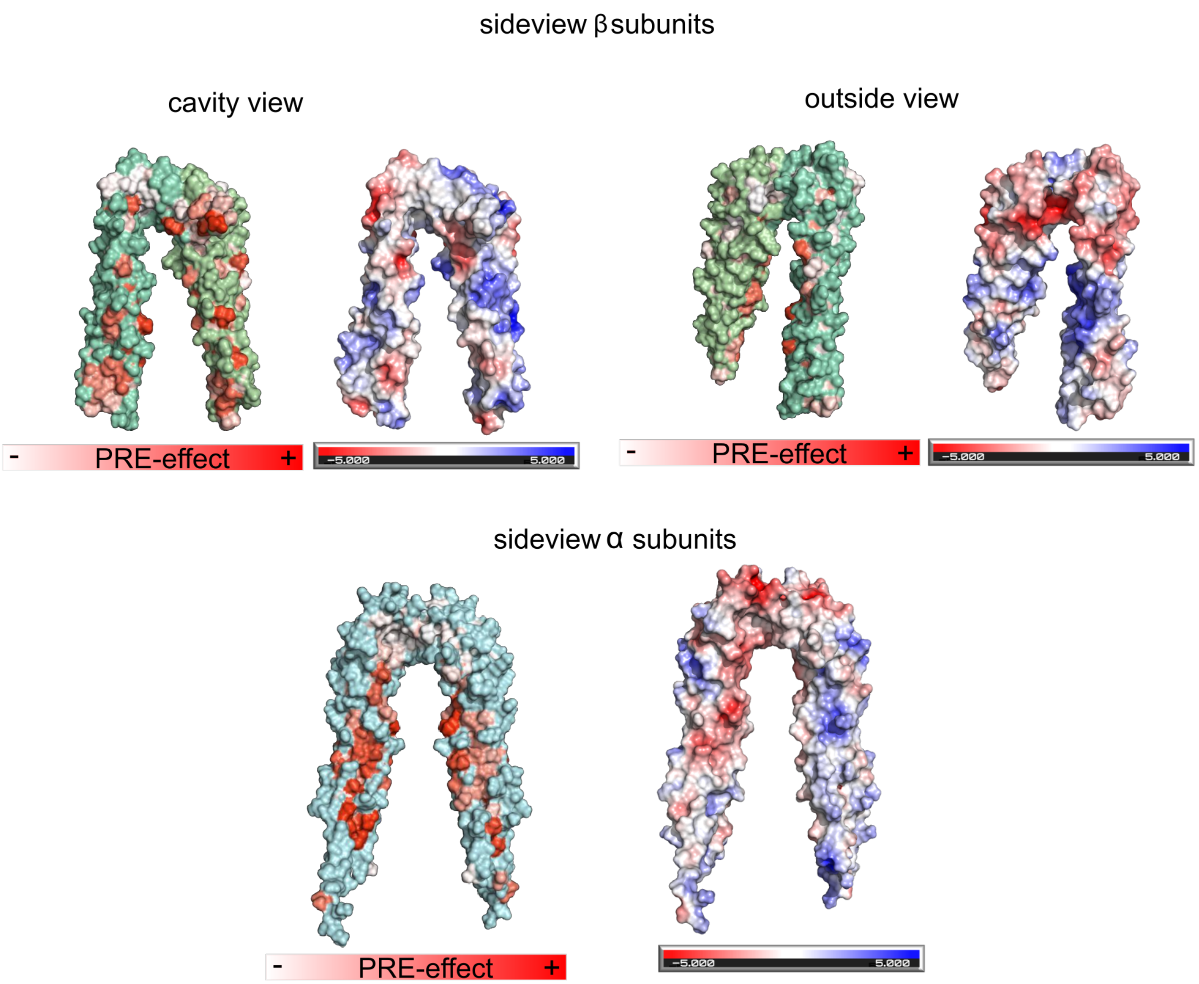

Supplementary Figure 6: Comparison of PRE-mapping and electrostatic surface on PhPFD. The interaction surface mapped on PhPFD by paramagnetic relaxation enhancement juxtaposed with the theoretical electrostatic surface computed by APBS electrostatics implemented in pymol (Baker et al. Electrostatics of nanosystems: Application to microtubules and the ribosome. *Proceedings of the National Academy of Sciences of the United States of America* **98**, 10037–10041 (2001)).

a

	Vector (antibiotic resistance)	Sequence
**PFD1 (beta)** **14.2 kDa**	**pET-21a** **(Ampicillin)**	MAAPVDLELK KAFTELQAKV IDTQQKVKLA DIQIEQLNRT KKHAHLTDTE IMTLVDETNM YEGVGRMFIL QSKEAIHSQL LEKQKIAEEK IKELEQKKSY LERSVKEAED NIREMLMARR AQ
**PFD2 (beta)** **16.9 kDa** **with tag 18.8 kDa**	**pET-41** **(Kanamycin)**	MGSSHHHHHH SSGLVPRGSH MAENSGRAGK SSGSGAGKGA VSAEQVIAGF NRLRQEQRGL ASKAAELEME LNEHSLVIDT LKEVDETRKC YRMVGGVLVE RTVKEVLPAL ENNKEQIQKI IETLTQQLQA KGKELNEFRE KHNIRLMGED EKPAAKENSE GAGAKASSAG VLVS
**PFD3 (alpha)** **18.7 kDa**	**pET-21a** **(Ampicilin)**	MKQPGNETAD TVLKKLDEQY QKYKFMELNL AQKKRRLKGQ IPEIKQTLEI LKYMQKKKES TNSMETRFLL ADNLYCKASV PPTDKVCLWL GANVMLEYDI DEAQALLEKN LSTATKNLDS LEEDLDFLRD QFTTTEVNMA RVYNWDVKRR NKDDSTKNKA
**PFD4 (beta)** **15.2 kDa** **with tag 17.1 kDa**	**PET-41** **(Kanamycin)**	MGSSHHHHHH SSGLVPRGSH MKKAAAEDVN VTFEDQQKIN KFARNTSRIT ELKEEIEVKK KQLQNLEDAC DDIMLADDDC LMIPYQIGDV FISHSQEETQ EMLEEAKKNL QEEIDALESR VESIQRVLAD LKVQLYAKFG SNINLEADES
**PFD5 (alpha)** **17.3 kDa**	**pET-21a** **(Ampicillin)**	MAQSINITEL NLPQLEMLKN QLDQEVEFLS TSIAQLKVVQ TKYVEAKDCL NVLNKSNEGK ELLVPLTSSM YVPGKLHDVE HVLIDVGTGY YVEKTAEDAK DFFKRKIDFL TKQMEKIQPA LQEKHAMKQA VMEMMSQKIQ QLTALGAAQA TAKA
**PFD6 (beta)** **14.6 kDa**	**pET-21a** **(Ampicillin)**	MAELIQKKLQ GEVEKYQQLQ KDLSKSMSGR QKLEAQLTEN NIVKEELALL DGSNVVFKLL GPVLVKQELG EARATVGKRL DYITAEIKRY ESQLRDLERQ SEQQRETLAQ LQQEFQRAQA AKAGAPGKA

b

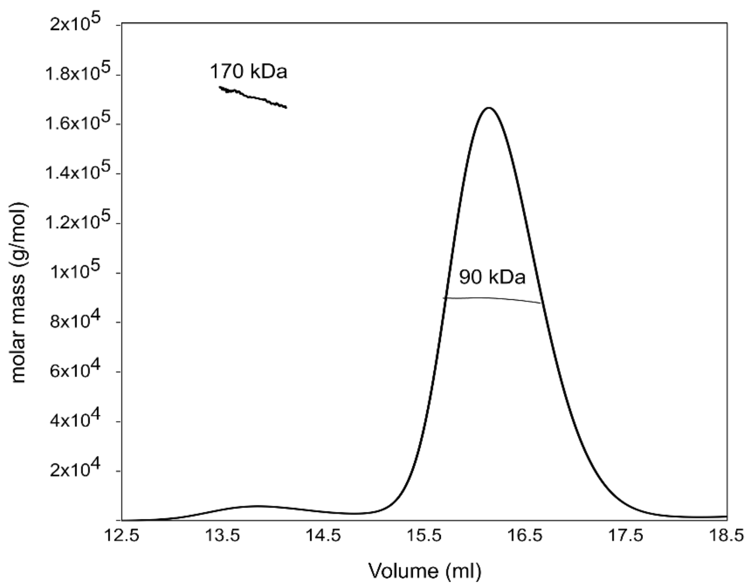

c

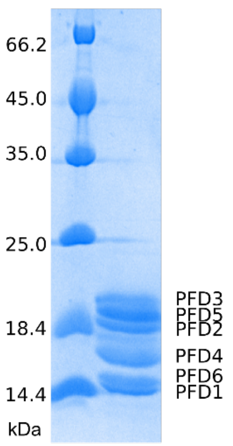

Supplementary Figure 7: Production of hPFD samples and quality control. a Summary of information on hPFD subunit sequences (PFD2 and PFD4 include polyhistidine tag sequences) and plasmids used in this study. **b** SEC-MALS analysis of heterohexameric hPFD sample. SEC-MALS analysis showed two species, a minor species at about 170 kDa (~5%) and a major one at about 90 kDa (~95%). The formation of two species, the hexameric complex and a complex of double size was already observed by Aikawa et al.[65] **c** SDS-PAGE of sample after purification. On the SDS-PAGE six bands appear, confirming the formation of a pure hPFD complex.

a

	Vector (antibiotic resistance)	Sequence
β-subunit 13.3 kDA	pET23c (Ampicillin)	MQNIPPQVQA MLGQLDTYQQ QLQLVIQQKQ KVQADLNEAK KALEEIETLP DDAQIYKTVG TLIVKTTKEK AVQELKEKI ETLEVRLNAL NRQEQKINEK VKELTQKIQA ALRPPTAG
α-subunit 16.6 kDa	pET23c (Ampicillin)	MAQNNKELEK LAYEYQVLQA QAQILAQNLE LLNLAKAEVQ TVRETLENLK KIEEEKPEIL VPIGAGSFLK GVIVDKNNAI VSVGSGYAVE RSIDEAIGFL EKRLKEYDEA IKKTQGALAE LEKRIGEVAR KAQEVQQKQS MTSFKVKK

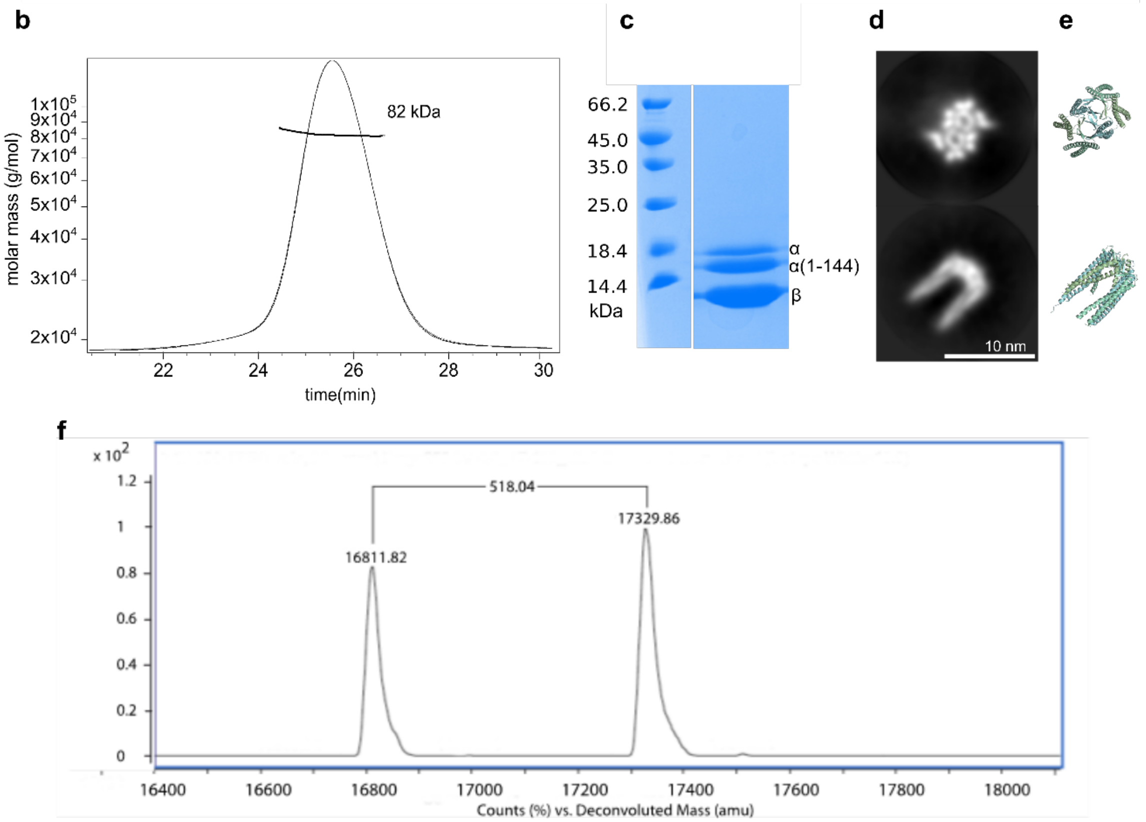

Supplementary Figure 8: PhPFD production and quality control. a Summary of information on PhPFD α and β subunits sequences and plasmids used in this study. **b** SEC-MALS analysis of $\alpha_2\beta_4$–PhPFD sample. The mass of PhPFD was estimated to be 82 kDa. **c** SDS-PAGE analysis and subsequent mass spectrometry analysis (f) indicated the presence of a partial cleavage on the C-terminal residues 145-148 of the α-subunit. This cleavage located in the unstructured C-terminal sequence does not hinder the formation of the complex, as can be observed from (**d**) the 2D classes obtained from cryo-EM analysis. Formation of the β-barrel structures with the six pairs of protruding helices can be clearly seen from the top view. Formation of the β-coiled coil α-helices is also observed in the side view. The other side view is missing due to preferential orientation hampering determination of 3D structures from cryo-EM images. In (**e**) the according views of the molecular model are shown. In (**f**) mass spectrometry analysis showing cleavage on the C-terminal residues 145-148 of the α-subunit. The mass difference of 518 amu correspond to the mass of the KVKK fragment (483.66 + 32 for the 32 deuterium + 1 for the ^{13}C on one of the methyl group of the valine).

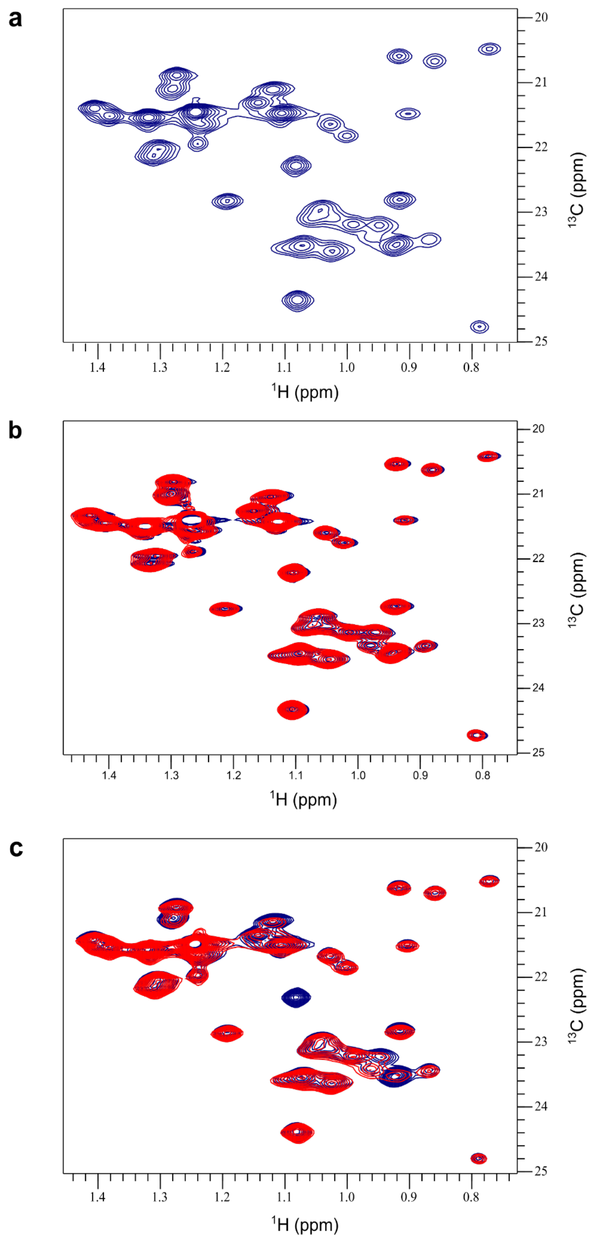

Supplementary Figure 9: Interaction between paramagnetically labelled IAPP and PhPFD. a Reference $^{13}CH_3$-TROSY spectrum of PhPFD labelled on methyl residues on the β subunits at 30 °C in 25mM MES/NaOH (pH 6.5), 25mM $MgCl_2$. **b** PhPFD control spectra in presence of 1:1 of Lu^{3+}-loaded DOTA (blue) or Gd^{3+}-loaded DOTA (red). **c** PRE effect detected on $^{13}CH_3$-labelled PhPFD in presence of diamagnetic Lu-IAPP (blue) or paramagnetic Gd-IAPP (red).

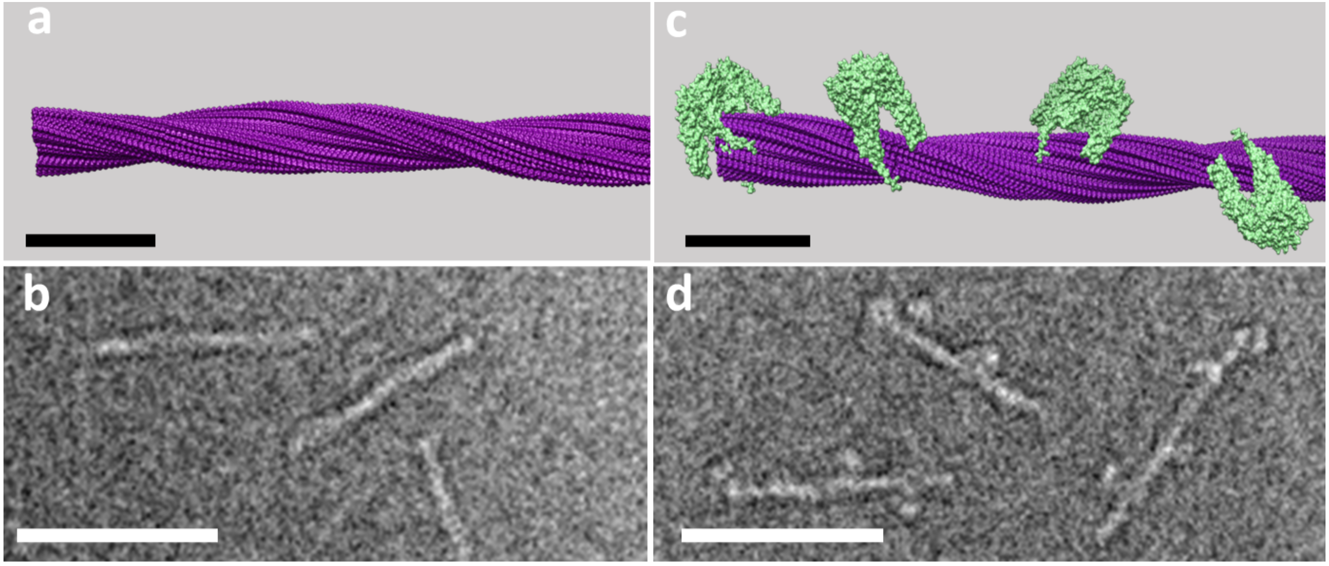

Supplementary Figure 10: Simulated EM images of PhPFD bound to IAPP fibrils surfaces and ends. Transmission electron microscopy images were simulated with TEM-simulator (Rullgård et al. Simulation of transmission electron microscope images of biological specimens. *Journal of Microscopy* **243**, 234–256 (2011). http://tem-simulator.sourceforge.net/), using the model shown in Fig. 4i. **a** Structure of the ordered part (residues 13-37) of polymorph 1 IAPP fibril. **b** Simulated TEM image of IAPP fibrils according to the structure depicted in (**a**). **c** Model of interaction between IAPP fibril and PhPFD. **d** Simulated TEM image of IAPP fibril decorated with PhPFD. Horizontal black and white bars correspond to a length of 10 nm and 50 nm, respectively.

a

b

c

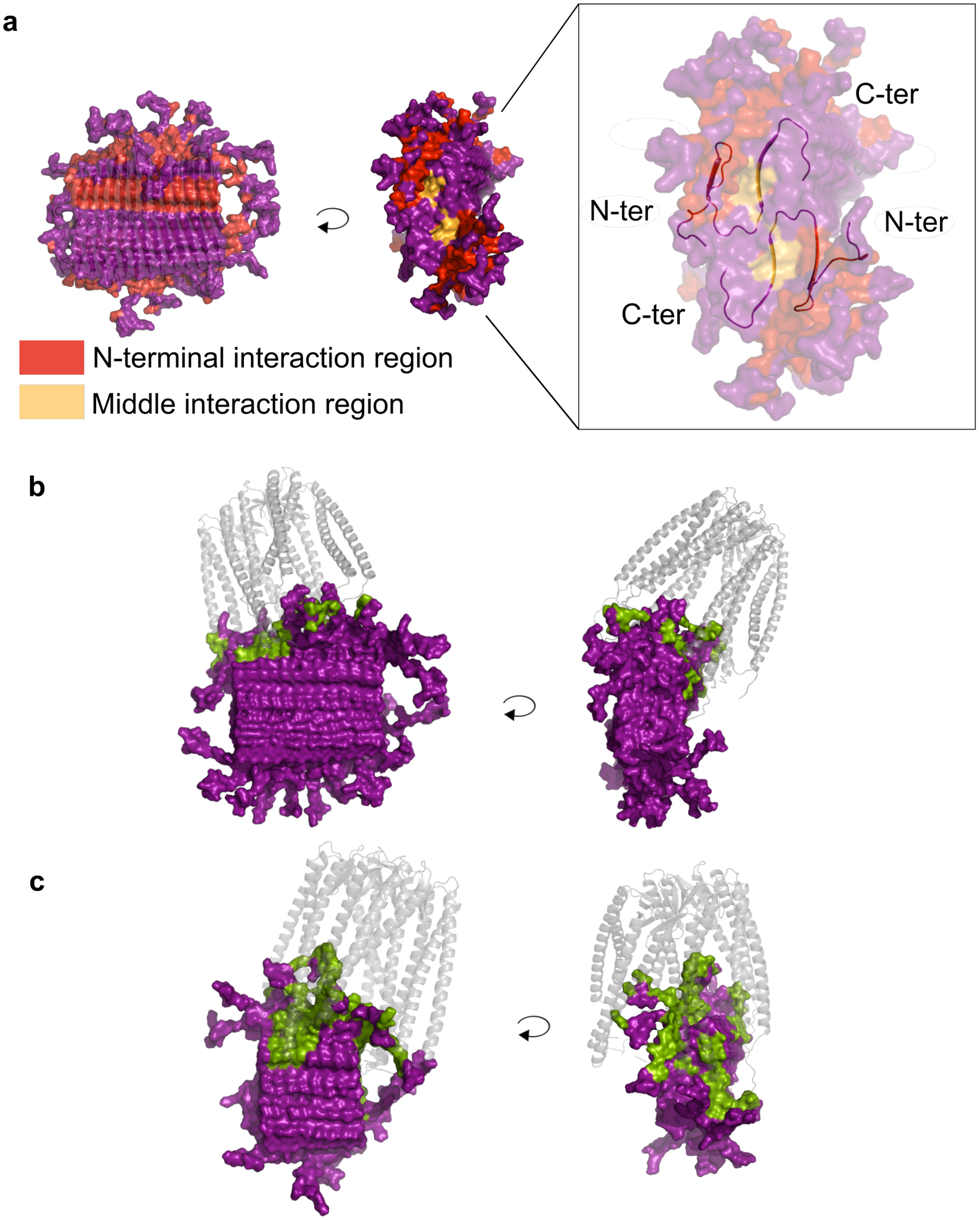

Supplementary Figure 11: Representation of residues of IAPP on the fibril surface or on the fibril ends interacting with PFD. a Amino acids used as docking restraints (see Methods section) are colour-coded in red (N-terminal binding segment) and yellow (middle interacting segment). Panel (**b**) presents in green the amino acids of the IAPP fibril surface/PFD docking model that are distant by less than 6 Å from PFD residues. **c** Same as (**b**) with fibril end/PFD docking model.
