# Supplementary material for: Structural basis for the inhibition of IAPP fibril formation by the co-chaperonin prefoldin
Source: Nat Commun. 2022 May 2;13:2363. doi: 10.1038/s41467-022-30042-y (PMC9061850; doi:10.1038/s41467-022-30042-y)
Supplement: Supplementary file 3 — Reporting Summary [file 41467_2022_30042_MOESM3_ESM.pdf]

Corresponding author(s): Boisbouvier, Hoyer

Last updated by author(s): Mar 29, 2022

Reporting Summary

Nature Portfolio wishes to improve the reproducibility of the work that we publish. This form provides structure for consistency and transparency in reporting. For further information on Nature Portfolio policies, see our Editorial Policies and the Editorial Policy Checklist.

Statistics

For all statistical analyses, confirm that the following items are present in the figure legend, table legend, main text, or Methods section.

n/a	Confirmed	
☐	☒	The exact sample size (*n*) for each experimental group/condition, given as a discrete number and unit of measurement
☒	☐	A statement on whether measurements were taken from distinct samples or whether the same sample was measured repeatedly
☐	☒	The statistical test(s) used AND whether they are one- or two-sided<br>*Only common tests should be described solely by name; describe more complex techniques in the Methods section.*
☒	☐	A description of all covariates tested
☒	☐	A description of any assumptions or corrections, such as tests of normality and adjustment for multiple comparisons
☐	☒	A full description of the statistical parameters including central tendency (e.g. means) or other basic estimates (e.g. regression coefficient) AND variation (e.g. standard deviation) or associated estimates of uncertainty (e.g. confidence intervals)
☐	☒	For null hypothesis testing, the test statistic (e.g. *F*, *t*, *r*) with confidence intervals, effect sizes, degrees of freedom and *P* value noted<br>*Give P values as exact values whenever suitable.*
☒	☐	For Bayesian analysis, information on the choice of priors and Markov chain Monte Carlo settings
☒	☐	For hierarchical and complex designs, identification of the appropriate level for tests and full reporting of outcomes
☒	☐	Estimates of effect sizes (e.g. Cohen's *d*, Pearson's *r*), indicating how they were calculated

Our web collection on statistics for biologists contains articles on many of the points above.

Software and code

Policy information about availability of computer code

Data collection	For the collection of experimental data, the following softwares were used: topSpin 3.5; NMRlib2.0; EPU 1.8; Digital Micrographs; NanoWizard Control Software v.5 version 5.0.84 by JPK, Reader Control by BMG, BLItz Pro 1.2.1.5 by ForteBio.<br>All these softwares are described in published literature and available to the scientific community.
Data analysis	For the analysis of experimental data, the following softwares were used: nmrPipe9.4; relion3.08; ccpnmr analysis 2.4; TITAN; gwyddion2.59; HADDOKCK web server 2.4; MODELLER9.23, JPK Data Processing version spm-5.0.84.<br>All these softwares are described in published literature and available to the scientific community.<br>The custom-developed software used in this study is publicly available at Zenodo repository under accession code 6380983 [https://doi.org/10.5281/zenodo.6380983].

For manuscripts utilizing custom algorithms or software that are central to the research but not yet described in published literature, software must be made available to editors and reviewers. We strongly encourage code deposition in a community repository (e.g. GitHub). See the Nature Portfolio guidelines for submitting code & software for further information.

Data

Policy information about availability of data

All manuscripts must include a data availability statement. This statement should provide the following information, where applicable:

- Accession codes, unique identifiers, or web links for publicly available datasets
- A description of any restrictions on data availability
- For clinical datasets or third party data, please ensure that the statement adheres to our policy

The NMR assignments data together with corresponding NMR experiments used in this study are available in the Biological Magnetic Resonance Data Bank under

accession code 51259 [https://bmrb.io/data_library/summary/?bmrbId=51259] for IAPP(-> see ref 87) and 50845 [https://bmrb.io/data_library/summary/?bmrbId=50845] for PhPFD (-> ref 88). The titration and PRE NMR experiments used in this study are available in the Biological Magnetic Resonance Data Bank under accession code bmrbig32 [https://bmrbig.org/released/bmrbig32]. All other experimental data (EM, AFM, BLI, ThT and Cell viability assays) used in this study are available in Zenodo repository (-> ref 89) under accession code 6319386 [https://zenodo.org/record/6319386].
3D structures previously deposited in the PDB were used for this study and the corresponding accession codes (2ZDI,2L86, 6Y1A accompagnied with hyperlinlinks are provided in the text and figure legends.

Field-specific reporting

Please select the one below that is the best fit for your research. If you are not sure, read the appropriate sections before making your selection.

- [x] Life sciences
- [ ] Behavioural & social sciences
- [ ] Ecological, evolutionary & environmental sciences

For a reference copy of the document with all sections, see nature.com/documents/nr-reporting-summary-flat.pdf

Life sciences study design

All studies must disclose on these points even when the disclosure is negative.

Sample size	No statistical methods were used to predetermine the sample size. Sample sizes for the experiments were chosen based on literature analysis and on previous experience with similar setups that showed significance. Sample size is described for each experiment in the corresponding figure legend.
Data exclusions	No data were exluded from the analysis
Replication	ThT-fluorescence assays were performed in triplicates. In rare cases of not tight sealing, followed by drying out of the solution (0-4.4% of the number of samples per experiment), these single samples were excluded from the analysis which then was performed on duplicates. AFM and EM images were obtained for several areas on the substrate to ensure that the presented assembly types are representative for the respective sample. The MTT cell viability test was performed twice with four to five technical replicates per experiment. BLI measurements were repeated three times. All 2D NMR spectra were recorded at least twice to ensure reproducibility of the observed spectral changes All attempts to replicate the data were successful.
Randomization	Cells and samples were randomly assigned to different experimental conditions.
Blinding	No blinding was performed during data acquisition, because acquisition was performed after standard procedures in an unbiased manner.

Reporting for specific materials, systems and methods

We require information from authors about some types of materials, experimental systems and methods used in many studies. Here, indicate whether each material, system or method listed is relevant to your study. If you are not sure if a list item applies to your research, read the appropriate section before selecting a response.

Materials & experimental systems

n/a	Involved in the study	
x		Antibodies
	x	Eukaryotic cell lines
x		Palaeontology and archaeology
x		Animals and other organisms
x		Human research participants
x		Clinical data
x		Dual use research of concern

Methods

n/a	Involved in the study	
x		ChIP-seq
x		Flow cytometry
x		MRI-based neuroimaging

Eukaryotic cell lines

Policy information about cell lines

Cell line source(s)	RIN-m5F cells (rat pancreatic beta cells - ATCC, CRL-11605, Manassas, VA, USA)
Authentication	The RIN-m5F cells were not authenticated since they were newly ordered just for the cell culture experiment described in this study.
Mycoplasma contamination	The RIN-m5F cells were newly ordered just for the cell culture experiment described in this study and were tested negative by the supplier for mycoplasma contamination.
Commonly misidentified lines (See ICLAC register)	No commonly misidentified cell lines were used in this study.
